# Prognostic influence of mechanical cardiopulmonary resuscitation on survival in patients with out-of-hospital cardiac arrest undergoing ECPR on VA-ECMO

**DOI:** 10.3389/fcvm.2023.1266189

**Published:** 2024-01-11

**Authors:** A. Springer, A. Dreher, J. Reimers, L. Kaiser, E. Bahlmann, H. van der Schalk, P. Wohlmuth, N. Gessler, K. Hassan, J. Wietz, B. Bein, T. Spangenberg, S. Willems, S. Hakmi, E. Tigges

**Affiliations:** ^1^ Department of Cardiology and Critical Care, Asklepios Clinic St. Georg, Hamburg, Germany; ^2^ Asklepios ProResearch, Hamburg, Germany; ^3^ DZHK (German Centre for Cardiovascular Research), Partner Site Hamburg/Kiel/Luebeck, Hamburg, Germany; ^4^ Department of Cardiac Surgery, Asklepios Clinic St. Georg, Hamburg, Germany; ^5^ Department of Emergency Medicine, Asklepios Clinic St. Georg, Hamburg, Germany; ^6^ Department of Anaesthesiology and Critical Care, Asklepios Clinic St. Georg, Hamburg, Germany; ^7^ Department of Cardiology and Critical Care, Asklepios Clinic Altona, Hamburg, Germany; ^8^ Semmelweis-University, Budapest, Hungary

**Keywords:** ECPR, VA-ECMO, OHCA, ACCD, CPR

## Abstract

**Introduction:**

The use of venoarterial extracorporeal membrane oxygenation (VA-ECMO) in extracorporeal cardiopulmonary resuscitation (ECPR) in selected patients after out-of-hospital cardiac arrest (OHCA) is an established method if return of spontaneous circulation cannot be achieved. Automated chest compression devices (ACCD) facilitate transportation of patients under ongoing CPR and might improve outcome. We thus sought to evaluate prognostic influence of mechanical CPR using ACCD in patients presenting with OHCA treated with ECPR including VA-ECMO.

**Methods:**

We retrospectively analyzed data of 171 consecutive patients treated for OHCA using ECPR in our cardiac arrest center from the years 2016 to 2022. A Cox proportional hazards model was used to identify characteristics related with survival.

**Results:**

Of the 171 analyzed patients (84% male, mean age 56 years), 12% survived the initial hospitalization with favorable neurological outcome. The primary reason for OHCA was an acute coronary event (72%) followed by primary arrhythmia (9%) and non-ischemic cardiogenic shock (6.7%). In most cases, the collapse was witnessed (83%) and bystander CPR was performed (83%). The median time from collapse to VA-ECMO was 81 min (Q1: 69 min, Q3: 98 min). No survival benefit was seen for patients resuscitated using ACCD. Patients in whom an ACCD was used presented with overall longer times from collapse to ECMO than those who were resuscitated manually [83 min (Q1: 70 min, Q3: 98 min) vs. 69 min (Q1: 57 min, Q3: 84 min), *p* = 0.004].

**Conclusion:**

No overall survival benefit of the use of ACCD before ECPR is established was found, possibly due to longer overall CPR duration. This may arguably be because of the limited availability of ACCD in pre-clinical paramedic service at the time of observation. Increasing the availability of these devices might thus improve treatment of OHCA, presumably by providing efficient CPR during transportation and transfer.

## 1 Introduction

Out-of-hospital cardiac arrest (OHCA) continues to be one of the leading causes of death in Europe, with an incidence of 35 per 100,000 person-years and overall poor survival rates (9%) (1). Implementation of extracorporeal cardiopulmonary resuscitation (ECPR) utilizing venoarterial extracorporeal membrane oxygenation (VA-ECMO) has shown promising improvement of long-term outcome in selected patients (2, 3). Nonetheless, the performance of high-quality and uninterrupted cardiopulmonary resuscitation (CPR) prior to ECPR initiation seems to be crucial (4).

Current expert opinion states that ECPR should be established <60 min (min) after patient collapse, to achieve improved outcome (5). Furthermore, overall shorter time to implementation of CPR measures (no-flow time), as well as bystander witnessed collapse have consistently shown to have a beneficial impact on outcome (6). This calls for optimization of preclinical CPR quality.

Traditional manual CPR is the established foundation of contemporary life support, while effectiveness in achieving return of spontaneous circulation, as well as improvement of long-term outcomes remain limited (7, 8). In recent years, there has been a growing interest in the use of automated mechanical chest compression devices (ACCD) with small sample studies suggesting improved hemodynamics and long-term outcomes, especially in transfer and transport scenarios (4, 9, 10). This benefit could not be confirmed in a larger randomized controlled trial by Wik et al., stating non-inferiority of the use of ACCD only when used by experienced operators (11). The meta-analyses of the current literature support the non-superiority of ACCD compared with manual CPR, while also highlighting the effectiveness of mechanical CPR in transfer and transport settings (12, 13).

The aim of this study was to determine whether the use of ACCD prior to implementation of ECPR might increase overall and neurologically favorable survival in patients after OHCA.

## 2 Methods

### 2.1 Patient selection and statistical analyses

We report retrospective data from a single-center registry of patients treated with ECPR for refractory OHCA in the Cardiac Arrest Center (CAC) of the Asklepios Clinic St. Georg (Hamburg, Germany) between January 2016 and December 2022. In the studied time period, a total of 377 patients were treated with VA-ECMO in our center. In this analysis, we included 171 patients with OHCA for whom data on whether an ACCD was used were available. Therefore, we excluded a total of 206 patients with either a different indication for VA-ECMO therapy (e.g., cardiogenic shock without cardiac arrest, intra-hospital cardiac arrest) or insufficient available data on whether an ACCD was used prior to ECPR initiation. The remaining cases were categorized into two groups based on whether an ACCD (*n* = 146, ACCD) or manual CPR (*n* = 25, no ACCD) was used prior to ECPR. The two groups were characterized and the baseline characteristics were compared using Fisher’s exact test as well as Wilcoxon’s rank sum test when applicable. The comparison was followed up with a survival analysis using Kaplan–Meier analyses and a Cox proportional hazards model. Statistical analyses were performed using R Core Team (Vienna, Austria, 2023).

Primary outcome was defined as survival of the primary hospital admission with favorable neurological outcome [cerebral performance category (CPC) score ≤2]. Secondary outcome was defined as survival of the primary hospital admission, regardless of the CPC scoring. The study protocol was approved by the local ethics committee.

### 2.2 ECPR program

Embedded in a tertiary care hospital in the urban area of Hamburg (Germany) our CAC falls back on a long-term experience with VA-ECMO implantation and management with a focus on ECPR. We offer an around-the-clock ECMO service with a specialized intensive care unit, as well as specialized heart failure and chest pain units.

In case of OHCA patients arriving at the emergency department, the interdisciplinary cardiac arrest receiving team (CART) is alerted beforehand. The CART comprises personnel from the Departments of Interventional Cardiology, Anaesthesiology, and Emergency Medicine. Based on the presumed etiology, other departments are alerted simultaneously. Treatment decision is made upon arrival at a “cardiac arrest fast assessment area” based on the current expert opinion as well as the individual criteria with a focus on the avoidance of time delay. Positive indicators for ECPR initiation are witnessed collapse, performance of bystander CPR, no-flow time <5 min, low-flow time <60 min, age <75 years, as well as shockable initial rhythm. However, the final decision is left to the CART and no strict criteria for deferral are provided. VA-ECMO implantation is performed in the cardiac catheter laboratory under fluoroscopic guidance. In the absence of contraindications, uni- or bilateral peripheral femoral access is used and cannulation performed using Seldinger's technique. To prevent peripheral limb ischemia, the standard practice is the implantation of a distal perfusion cannula when feasible. Postinterventional further diagnostics include a coronary angiography, as well as an individualized computed tomography. Intensive care management is at the discretion of the intensive care specialist, following the current guidelines.

## 3 Results

### 3.1 Baseline characteristics

The studied collective was predominantly male (84%) and the mean age was 56 years. Overall prevalence of cardiovascular risk factors was high but did not differ significantly between the two groups (Table 1). The majority of the studied patients were resuscitated using an ACCD (85%).

**Table 1:** Baseline characteristics.

	All *n* = 171	No ACCD *n* = 25	ACCD *n* = 146	*p*-value^a^
Age (years)				0.28
Mean (SD)	56 (13)	58 (13)	56 (13)	
Gender, *n* (%)				0.08
Male	144 (84)	18 (72)	126 (86)	
Height (m)				0.15
Mean (SD)	1.76 (0.07)	1.74 (0.07)	1.77 (0.06)	
Missing (*N*)	39	6	33	
Weight (kg)				0.41
Mean (SD)	86 (18)	86 (29)	86 (16)	
Missing (*N*)	39	6	33	
BMI (kg/m^2^)				0.47
Mean (SD)	27.8 (5.6)	28.5 (9.7)	27.7 (4.6)	
Missing (*N*)	39	6	33	
Hypertension, *N* (%)	55 (48)	55 (12)	46 (43)	0.48
Missing (*N*)	56	3	53	
Diabetes (type 1 and 2), *N* (%)	23 (27)	7 (33)	20 (21)	0.25
Missing (*N*)	53	4	49	
Hyperlipoproteinemia, *N* (%)	22 (20)	6 (29)	16 (18)	0.36
Missing (*N*)	61	4	57	
Nicotine abuse, *N* (%)	39 (37)	7 (35)	32 (37)	0.85
Missing (*N*)	65	5	60	
Coronary artery disease, *N* (%)	119 (71)	18 (75)	101 (71)	0.66
Missing (*N*)	4	1	3	
Peripheral artery disease, *N* (%)	14 (11)	2 (9.1)	12 (11)	>0.99
Missing (*N*)	38	3	35	
Atrial fibrillation, *N* (%)	18 (14)	2 (9.1)	16 (15)	0.74
Missing (*N*)	45	3	42	
Any cardiomyopathy, *N* (%)	38 (28)	8 (36)	30 (26)	0.34
Missing (*N*)	35	3	32	
Chronic pulmonary disease, *N* (%)	3 (2.4)	0 (0)	3 (2.9)	0.99
Missing (*N*)	44	3	41	
Prior pulmonary embolism, *N* (%)	1 (0.8)	1 (4.8)	0 (0)	0.17
Missing (*N*)	48	4	44	
Chronic kidney disease, *N* (%)	11 (8.2)	2 (9.1)	12 (11)	>0.99
Missing (*N*)	37	3	34	
Prior percutaneous coronary intervention (PCI), *N* (%)	20 (13)	4 (17)	16 (12)	0.50
Missing (*N*)	16	2	14	
Prior operative coronary bypass grafting (CABG), *N* (%)	12 (7.5)	2 (8.7)	10 (7.3)	0.68
Missing (*N*)	11	2	9	
Prior non-coronary cardiac surgery, *N* (%)	3 (1.9)	1 (4.3)	2 (1.5)	0.38
Missing (*N*)	14	2	12	

^a^ Wilcoxon rank sum test or Fisher's exact test.

### 3.2 Preclinical data

Compared with manually resuscitated patients, patients in whom an ACCD was used showed a tendency to have a lower prevalence of witnessed collapse (82% vs. 88%, *p* = 0.57) and performance of bystander CPR (82% vs. 92%, *p* = 0.26) without attaining statistical significance. Overall mean time from collapse to implementation of advanced life support (ALS) measures [i.e. arrival of the emergency medical service (EMS)] and ECPR were significantly longer in the ACCD cohort [collapse to ALS: 8 min (Q1: 4 min, Q3: 12 min) vs. 5 min (Q1: 0 min, Q3: 8 min), *p* = 0.012; collapse to ECPR: 83 min (Q1: 70 min, Q3: 98 min) vs. 69 min (Q1: 57 min, Q3: 84 min), *p* = 0.004]. No relevant differences were found regarding the prevalence of shockable rhythm between the two groups (Table 2).

**Table 2:** Preclinical data.

	All *n* = 171	No ACCD *n* = 25	ACCD *n* = 146	*p*-value^a^
Witnessed collapse, *N* (%)	134 (83)	22 (88)	112 (82)	0.57
Missing (*N*)	9	0	9	
Bystander CPR, *N* (%)	140 (83)	23 (92)	117 (82)	0.26
Missing (*N*)	3	0	3	
Initial ECG, *N* (%)				0.61
PEA, asystole	77 (44)	8 (29)	69 (46)	
VT/VF	94 (56)	17 (71)	77 (54)	
Missing (*N*)	4	1	3	
Time from collapse to ALS (min)				0.012
Median [Q1, Q3]	8 [2, 11]	5 [0, 8]	8 [4, 12]	
Missing (*N*)	22	2	20	
Time from collapse to ECPR (min)				0.004
Median [Q1, Q3]	81 [69, 98]	69 [57, 84]	83 [70, 98]	
Missing (*N*)	29	5	24	

PEA, pulseless electric activity; VT, ventricular tachycardia; VF, ventricular fibrillation.

^a^ Wilcoxon rank sum test or Fisher's exact test.

### 3.3 Procedural data and complications

The primary reason for cardiac arrest in the studied collective was an acute coronary event (72%) followed by primary arrhythmia (9%) and non-ischemic cardiogenic shock (6.7%). In 19.4% of the cases an additional left ventricular venting device (Impella 2.5, Impella CP) was implanted in the primary procedure. No relevant differences were noted between the groups on this score. The median time from door to running ECPR was 15 min regardless of the use of ACCD (Table 3A).

**Table 3:** (A) Procedural data and (B) complications.

	All *n* = 171	No ACCD *n* = 25	ACCD *n* = 146	*p*-value^a^
(A)				
Door to ECMO time (min)				0.96
Median [Q1, Q3]	15 [13, 23]	15 [13, 22]	15 [12, 23]	
Missing (*N*)	18	3	15	
Left ventricular venting device, *N* (%)				0.39
Impella 2.5	17 (10)	4 (16)	13 (9)	
Impella CP	16 (9.4)	1 (4)	15 (10)	
None	137 (81)	20 (80)	117 (81)	
Missing (*N*)	1	0	1	
Diagnosis after procedure, *N* (%)				0.40
Acute coronary event	96 (72)	17 (77)	79 (71)	
Primarily arrhythmogenic	12 (9)	1 (4)	11 (9.8)	
Non-ischemic cardiogenic shock	9 (6.7)	0 (0)	9 (8)	
Takotsubo cardiomyopathy	2 (1.5)	0 (0)	2 (1.8)	
Aortic dissection	6 (4.5)	2 (9.1)	4 (3.6)	
Pulmonary embolism	7 (5.2)	1 (4.5)	6 (5.4)	
Hypothermia	2 (1.5)	1 (4.5)	1 (0.9)	
(B)				
Visceral laceration, *N* (%)	3 (1.8)	1 (4)	2 (1.4)	0.39
Missing (*N*)	3	0	3	
Hemothorax, *N* (%)	8 (4.7)	3 (12)	5 (3.5)	0.10
Missing (*N*)	2	0	2	
Relevant bleeding, *N* (%)	25 (15)	3 (12)	22 (15)	>0.99
Missing (*N*)	2	0	2	
Complications requiring surgical or interventional measures, *N* (%)	17 (10)	4 (16)	13 (9)	0.28
Missing (*N*)	1	0	1	
Hypoxic brain injury/cerebral edema, *N* (%)	32 (19)	3 (11)	29 (21)	0.34
Missing (*N*)	1	0	1	

^a^ Fisher's exact test.

Relevant periprocedural bleeding complications appeared in 15% of the ECPR cases, warranting further surgical or interventional measures in 10% of the cases. The prevalence of other complications such as visceral laceration due to CPR was low (1.8%) and showed no association with the use of ACCD. Cerebral hypoxemia and consecutive cerebral edema were recorded in 19% of cases, triggering the interdisciplinary end-of-life decision pathway. Prevalence of postprocedural hypoxic brain damage was numerically higher in the ACCD group (21%), without statistical difference to the manually resuscitated cohort (11%, Table 3B).

### 3.4 Outcome

**Figure 1:**
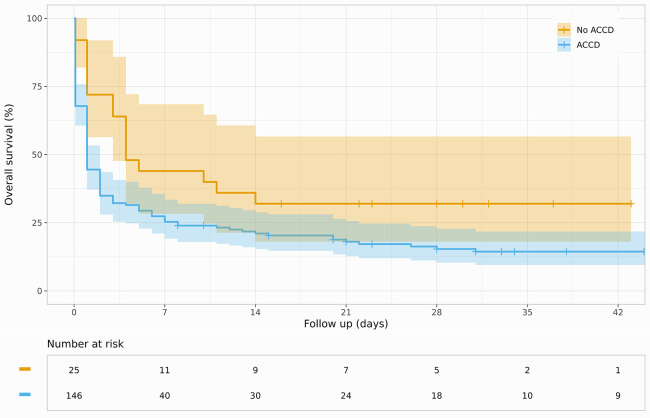
Kaplan–Meier curve for survival stratified by ACCD use.

**Figure 2:**
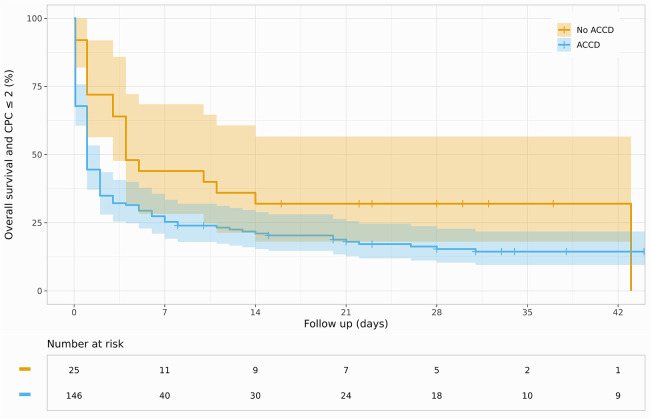
Kaplan–Meier curve for survival and favorable neurological outcome (CPC ≤ 2) stratified by ACCD use.

Overall, 18% of the studied patients survived the primary hospital stay, 12% with favorable neurological outcomes (CPC < 2). The comparison of the two groups demonstrated a numerical survival benefit among manually resuscitated patients [32% vs. 16% (ACCD); *p* = 0.087; Figure 1] with accordingly higher rates of favorable neurological outcomes [22% vs. 10% (ACCD); *p* = 0.16; Figure 2]. Interestingly, the surviving patients in the ACCD group showed a higher ratio of neurologically favorable survival compared with the surviving patients who were manually resuscitated [93% (ACCD) vs. 85%; *p* = 0.5]. Overall, 90% of the surviving patients were discharged with a favorable neurological outcome (Table 4).

**Table 4:** Outcomes.

	All *n* = 171	No ACCD *n* = 25	ACCD *n* = 146	*p*-value^a^
Survival of primary hospital admission, *N* (%)	31 (18)	8 (32)	23 (16)	0.087
Discharge CPC ≤2, *N* (%)	19 (12)	5 (22)	14 (10)	0.16
Missing (*N*)	11	2	9	
Proportion of CPC ≤2 in survivors, *N* (%)	19 (90)	5 (83)	14 (93)	0.50

^a^ Fisher's exact test.

### 3.5 Cox proportional hazards model

**Figure 3:**
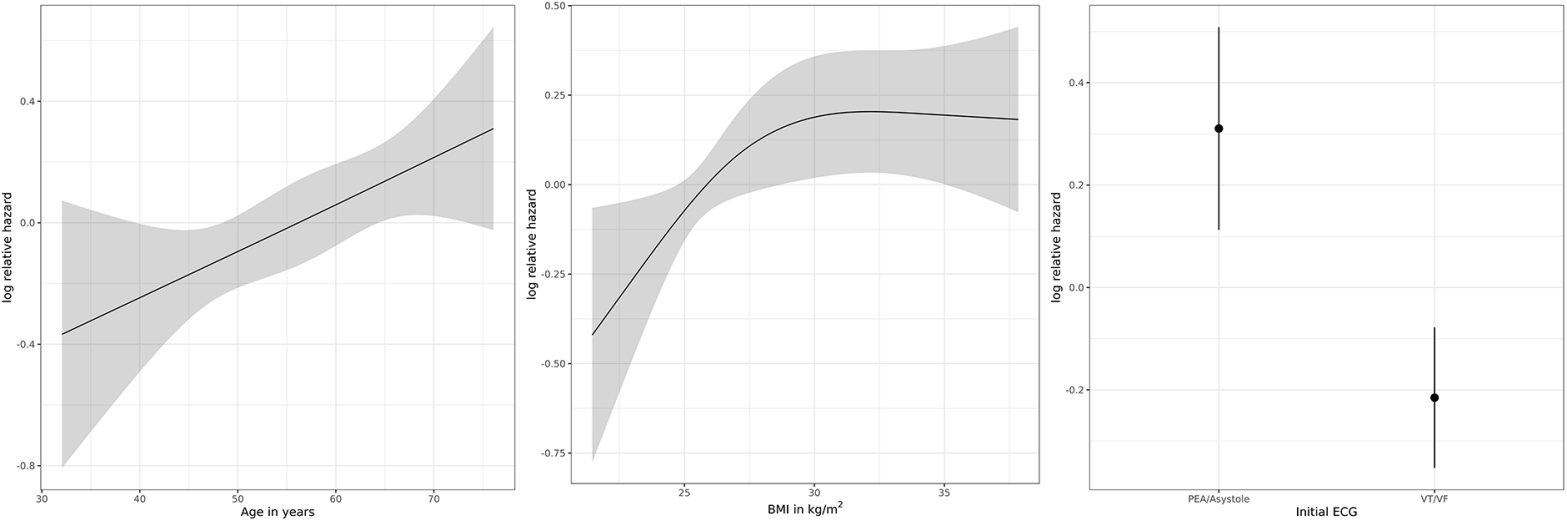
Logarithmic relative hazard for adverse outcome for age (in years), BMI (in kg/m^2^), and initial electrocardiographic (ECG) rhythm. PEA, pulseless electric activity; VT, ventricular tachycardia; VF, ventricular fibrillation.

The Cox proportional hazards model highlighted a higher age [hazard ratio (HR): 1.25; 95% confidence interval (CI): 1.03–1.52], a higher body mass index (HR: 1.36; 95% CI: 1.05–1.76), as well as an initially non-shockable rhythm (HR: 1.69; 95% CI: 1.21–2.37) as predictors for an adverse outcome (Figure 3).

## 4 Discussion

In this study, we characterized an all-comers collective of patients treated with ECPR for refractory OHCA confirming the known risk factors for an adverse outcome. Especially, the adverse impact of higher age and initial non-shockable rhythm were highlighted by the hazard model and are consistent with the current literature (14–16).

In contrast to our results, the impact of a higher body mass index (BMI) on adverse outcome is currently being discussed, with retrospective data suggesting no increase in mortality in obese patients undergoing ECPR (17). Nonetheless, patients with a higher BMI were noted to have overall longer resuscitation duration, therefore creating other powerful risk factors for an adverse outcome.

The use of ACCD was not associated with improved survival or neurologically favorable outcome in the context of ECPR, therefore confirming the results of contemporary meta-analyses and randomized controlled trials studying the effectiveness of ACCD in conventional CPR (11, 13). The use of ACCD was not associated with higher rates of complications, especially not visceral lacerations as described by the current literature (11).

We hypothesize that the driving factor for non-superiority in our collective might be the significantly longer resuscitation duration (from collapse to ECPR) as well as the longer time from collapse to initiation of ALS CPR measures in the cohort resuscitated with an ACCD. A prolonged resuscitation duration and therefore longer low-flow is broadly recognized as a driving risk factor for an adverse outcome in the context of ECPR (5, 16, 18, 19).

Arguably a possible explanation for the prolonged resuscitation times in the ACCD collective might be the late implementation of the extensive availability of ACCD devices in the area this investigation was set in. Prior to April 2021, ACCD had to be requested by the arriving EMS teams and separately transported to the collapse site in the urban area of Hamburg (Germany), thus potentially explaining a certain delay and therefore longer preclinical resuscitation duration. In addition, ACCDs are predominantly used in complex rescue scenarios, which alone might already be associated with prolonged prehospital CPR duration and possibly impaired CPR quality. This has to be taken into account when assessing our results. Nonetheless, our data show that even considering the mean resuscitation duration of >60 min, ECPR can improve survival compared with conventional CPR. These findings align with data questioning the exclusion of patients with resuscitation duration >60 min (20).

### 4.1 Limitations

Due to the retrospective nature of data collection from a single center, the applicability of the presented results to a general population might be limited. Portraying a comparatively long study time interval, our analysis has to be evaluated under the constraint of the inherent learning curve, not only regarding the management of ECPR patients, but also identification and selection of patients susceptible to this therapy. Lastly, driven by the high prevalence of ACCD usage in the displayed collective, manual CPR might be statistically underpowered in the comparison with mechanically assisted CPR.

### 4.2 Conclusion

In conclusion, our data confirmed the known risk factors for an adverse outcome in ECPR, although our results, as well as the current literature, emphasize the multifactorial impact of these risk factors. Basing the decision of whether a patient is suitable for ECPR, or excluding this therapeutic option, on just one adverse indicator should be carefully discussed. Regarding the use of ACCD, our findings support the current data for non-superiority of these devices compared with manual CPR. Further studies should address possible benefits of these devices in the context of transport and transfer situations.

## Data Availability

The raw data supporting the conclusions of this article will be made available by the authors, without undue reservation.
